# Diagnostic Value of Measuring Platelet Von Willebrand Factor in Von Willebrand Disease

**DOI:** 10.1371/journal.pone.0161310

**Published:** 2016-08-17

**Authors:** Alessandra Casonato, Maria Grazia Cattini, Viviana Daidone, Elena Pontara, Antonella Bertomoro, Paolo Prandoni

**Affiliations:** 1 Department of Medicine, University of Padua Medical School, Padua, Italy; 2 Department of Cardiologic, Thoracic and Vascular Sciences, University of Padua Medical School, Padua, Italy; Royal College of Surgeons in Ireland, IRELAND

## Abstract

Von Willebrand disease (VWD) may be caused by an impaired von Willebrand factor (VWF) synthesis, its increased clearance or abnormal function, or combinations of these factors. It may be difficult to recognize the different contributions of these anomalies. Here we demonstrate that VWD diagnostics gains from measuring platelet VWF, which can reveal a defective VWF synthesis. Measuring platelet VWF revealed that: severe type 1 VWD always coincided with significantly lower platelet and plasma VWF levels, whereas mild forms revealed low plasma VWF levels associated with low or normal platelet VWF levels, and the latter were associated with a slightly shorter VWF survival; type Vicenza (the archetype VWD caused by a reduced VWF survival) featured normal platelet VWF levels despite significantly reduced plasma VWF levels; type 2B patients could have either normal platelet VWF levels associated with abnormal multimer patterns, or reduced platelet VWF levels associated with normal multimer patterns; type 2A patients could have reduced or normal platelet VWF levels, the former associated mainly with type 2A-I, the latter with type 2A-II; plasma and platelet VWF levels were normal in type 2N, except when the defect was associated with a quantitative VWF mutation. Our findings show that measuring platelet VWF helps to characterize VWD, especially the ambiguous phenotypes, shedding light on the mechanisms underlying the disorder.

## Introduction

Von Willebrand factor (VWF) is a high-molecular-weight multimeric glycoprotein with a fundamental role in the early stages of hemostasis, promoting the binding of platelet VWF at the site of vascular injury [1]. It also serves as a carrier of FVIII [2]. VWF is found in plasma, platelets, megakaryocytes, endothelial cells and the subendothelial matrix, and it is synthesized by endothelial cells and megakaryocytes [3,4]. VWF synthesized in endothelial cells is either stored in Weibel Palade bodies or constitutively secreted into the plasma, which assures circulating VWF levels [4,5]. Platelet VWF synthesized by megakaryocytes accounts for 10% to 20% of all the VWF in the blood. It is secreted from the alpha granules at the site of vascular injury following platelet activation and during platelet plug formation [6]. Both plasma and platelet VWF are therefore involved in primary hemostasis.

Although definitive proof is still lacking, it is currently accepted that VWF is biosynthesized in megakaryocytes and endothelial cells in much the same way, but a number of important differences have emerged between platelet and plasma VWF. First of all, platelet VWF is richer in hemostatically active ultra-large VWF multimers [6], and it reveals no triplet pattern on multimer analysis [7], confirming the recent claim that platelet VWF is more resistant to ADAMTS13 proteolysis than plasma VWF [8]. The sialylation of N-linked glycans is also at least 50% lower for platelet VWF than for plasma VWF [7–9], and while there are O blood group structures on platelet VWF, there are no group A- or B-glycans [10,11]. ABO blood group consequently influences circulating VWF concentrations, but has no effect on platelet VWF levels [12,13].

VWF quantitative reductions and/or abnormalities lead to von Willebrand disease (VWD), which is probably the most common inherited bleeding disorder. Quantitative VWF defects cause VWD type 1 and type 3, while VWF functional abnormalities are responsible for type 2, which involves a defective interaction with platelets (types 2A, 2B and 2M) or FVIII (type 2N) [14]. The heterogeneity of the VWD phenotype also concerns platelet VWF content, which differs in the various types and subtypes of the disease [15,16]. Studies conducted in the past on type 1 VWD with both low and normal platelet VWF content demonstrated that the latter form is characterized by shorter bleeding times than the former [17]. This difference was used to distinguish type 1 VWD patients from those with other variants of the disease [17]. In type 3 VWD patients, on the other hand, combined infusions of cryoprecipitate and platelets can correct or considerably improve bleeding times, whereas cryoprecipitate infusion alone cannot [18]. The above findings are confirmed by the observation that pigs with severe VWD engrafted with normal bone marrow produce platelets with a normal VWF, and bleeding times become shorter than before the transplant, despite unchanged plasma VWF levels [19]. Other studies in pigs showed less conclusive results, however, and indicated that plasma VWF is the major determinant of bleeding time [20]. Taken together, these findings show that platelet VWF contributes to hemostasis, and that its role may vary within the heterogeneous setting of VWD.

Here we report on the platelet VWF content identified in different types of VWD, focusing on the value of measuring platelet VWF for the purpose of diagnosing VWD accurately and understanding the mechanisms underlying this disease.

## Materials and Methods

All subjects were studied after obtaining their written informed consent in accordance with the Helsinki Declaration, and the study was approved by our institutional review board (Ethics Committee of University of Padua and Padua Hospital; approval number 730Pt).

### Hemostatic tests

Blood samples were drawn from the antecubital vein and anticoagulated using 3.8% sodium citrate (1/10 vol/vol), which was supplemented with 60 mM N-ethylmaleimide (NEM), 50 mM EDTA and 200 kallikrein inhibitory units (KIU)/mL of aprotinin when blood samples were collected to assess platelet VWF content. Basic hemostatic analyses, i.e. plasma VWF antigen (VWF:Ag), VWF collagen binding (VWF:CB), VWF ristocetin cofactor (VWF:RCo), ristocetin-induced platelet aggregation (RIPA), VWF multimers and FVIII, were conducted as described elsewhere [21]. The capacity of VWF to bind FVIII (VWF:FVIIIB) was explored with an ELISA that uses recombinant FVIII (Baxter, Deerfield, Illinois) as exogenous FVIII [22]. DDAVP (1-desamino-8-D-arginine vasopressin; Emosint, Sclavo, Italy) was administered subcutaneously at a dose of 0.3 μg/kg. Blood samples were collected before and then 15, 30, 60, 120, 180, 240, 360 and 480 min, and 24 hours after administering DDAVP. The time courses of the VWF:Ag and VWF:CB plasma concentrations after DDAVP were analyzed using a one-compartment model with first-order input and output kinetics, as described elsewhere [23].

Platelet VWF:Ag was measured with the home-made ELISA used to measure plasma VWF [24]. Platelet-rich plasma (PRP) added with 3% EDTA in PBS buffer was centrifuged at 6800 x g for 45 seconds. After resuspension, platelets were washed twice, then resuspended at 1 x 10^6^/μL in PBS-EDTA and lysed with 1% Triton-X100. A pool of platelets obtained from 20 normal subjects, washed and adjusted to 1 x 10^6^ /uL, was used to prepare standard curves. The values were expressed in units per deciliter, taking the optical density in the first dilution of normal pooled platelets as 100. The normal range for platelet VWF (70–140 U/dL) was obtained by studying 45 normal subjects.

### Genetic analysis

Genomic DNA was extracted from peripheral blood leukocytes using the QIAamp DNA blood Mini Kit (QIAGEN, Hilden, Germany). All 52 exons of the VWF gene, including the intron-exon boundaries and the 3’ and 5’ regulation sites, were amplified and sequenced using primers chosen according to the VWF sequence identified by Mancuso et al [25].

### Statistical analysis

Laboratory data were expressed as means ± standard errors (SE). The t-test was used to compare all the results and a correlation analysis was conducted to assess the association between the parameters. Welch’s correction was used when variances were not equal. P values below 0.05 were considered statistically significant.

## Results

The study involved 156 patients with VWD: 72 had type 1 VWD, 5 had type 3, 16 had type Vicenza, 28 had type 2B, 14 had type 2A, and 21 had type 2N. All the type 2 VWD patients had a phenotypic diagnosis confirmed by genetic analyses.

### Type 1

Type 1 VWD was diagnosed on the strength of a homogeneous decrease in plasma VWF:Ag and function (VWF:RCo, VWF:CB and VWF:FVIIIB) in the absence of any evident VWF multimer structural abnormalities. Type 1 was divided into mild and severe forms depending on the circulating VWF levels: cases were considered mild for VWF levels below 50 U/dL, or severe for plasma VWF levels below 10 U/dL. Sixty-four patients had mild type 1 VWD and 8 were cases of severe type 1 VWD.

#### Mild type 1 VWD

**Fig 1:**
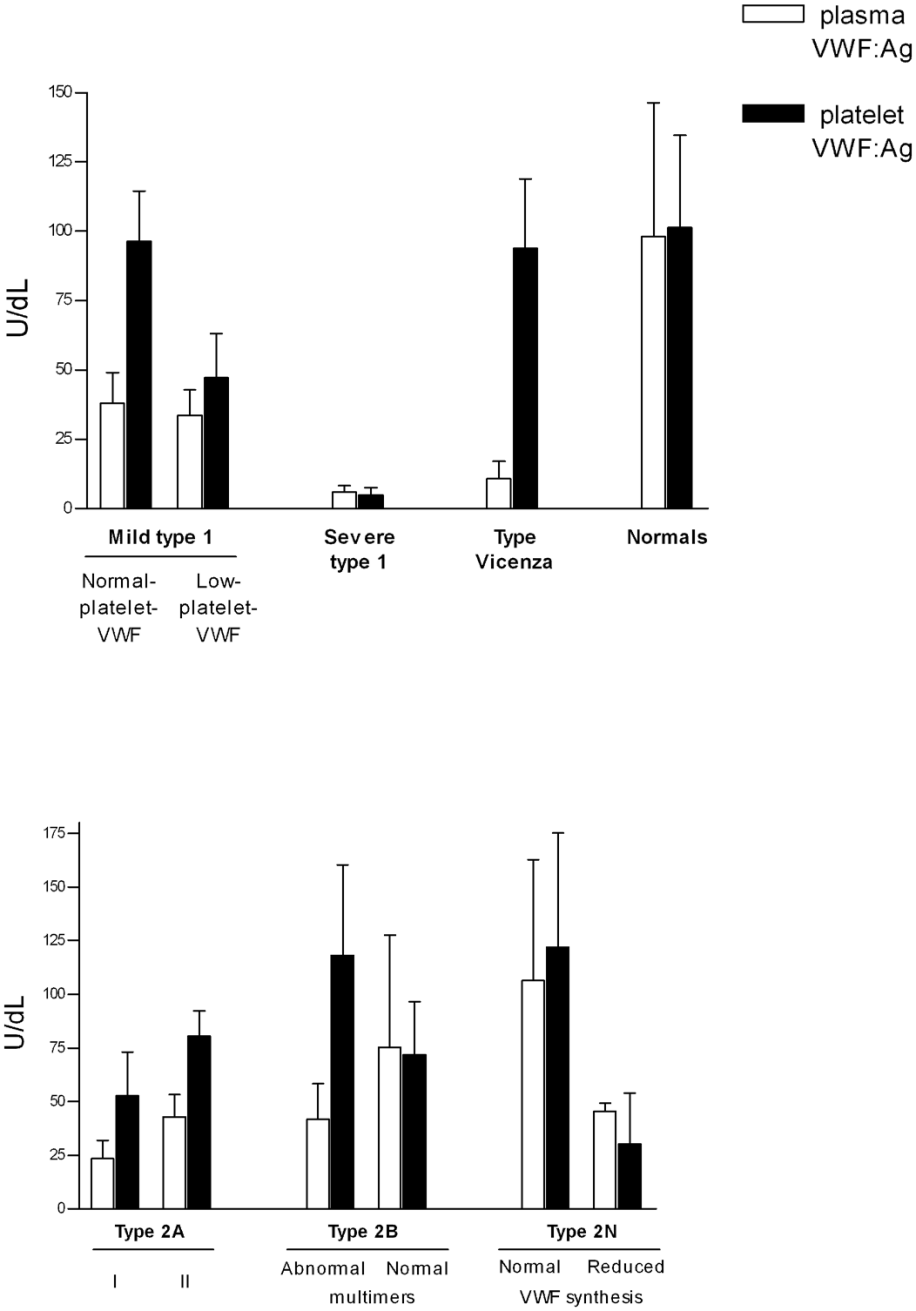
Mean plasma and platelet VWF in patients with quantitative (upper panel) and functional (lower panel) VWF defects. Type 1 VWD patients were divided into severe and mild cases, based on their circulating VWF levels (< 10U/dL and < 50 U/dL, respectively). Mild type 1 patients were further grouped according to their normal or low platelet VWF content. Functional VWF defects in type 2A were separated into a defective VWF synthesis (2A-I) and an increased susceptibility to ADAMTS13 (2A-II). Type 2B cases were divided according to their multimer pattern, and type 2N according to whether VWF synthesis was normal or reduced, the latter depending on the nature of the type 2N mutation, or the presence of a combined quantitative VWF mutation.

Among the cases of mild type 1 VWD, 20 patients had normal platelet VWF levels (96.4+/-4.0 U/dL [normal range 70–140 U/dL]) and 44 had a reduced platelet content (47.2+/-2.5 U/dL) (Table 1), but the multimer organization revealed no significant abnormalities in either instance. These patients are identified in the present paper as having *normal-platelet-VWF* or *low-platelet-VWF mild type 1 VWD*, respectively (Fig 1). The mean plasma VWF levels in these two groups were not dissimilar, i.e. 33.6+/-1.4 U/dL in low-platelet-VWF cases and 37.9+/2.5 U/dL in normal-platelet-VWF patients. The low-platelet-VWF mild type 1 patients showed a significant correlation between plasma and platelet VWF content (p<0.05). Some differences in the FVIII/VWF:Ag ratio emerged, since it appeared to be almost normal in normal-platelet-VWF mild type 1 VWD (1.3+/-0.1), but higher than normal in low-platelet-VWF patients (1.9+/-0.2), the normal range being 0.75–1.2 (Table 1).

**Table 1:** Main haemostatic findings of the VWD patients with quantitative VWF defects studied.

VWD type		No subjects/Families	VWF:Ag(U/dL)	VWF:CB(U/dL)	VWF:RCo(U/dL)	VWF:FVIIIB (U/dL)	FVIII(U/dL)	FVIII/VWF:Ag	Platelet VWF(U/dL)	BS
Mild type 1	Normal-platelet-VWF	20/8	37.9±11.2	35.2±13.3	35.9±11.6	33.9±10.8	47.4±14.1	1.3±0.3	96.4±18.0	4.8±4.1
	Low-platelet-VWF	44/32	33.6±9.2	32.0±11.2	31.8±11.0	32.2±10.5	56.8±21.9	1.9±1.1	47.2±15.9	3.8±3.4
Severe type 1		8/7	5.9±2.4	4.0±1.72	7.1±3.7	6.1±1.83	32.2±17.8	5.2±1.8	4.8±2.7	11.5±4.1
Type 3		5/3	ND	ND	ND	ND	4.5±2.2	ND	ND	24±4.8
Type Vicenza		16/10	10.7±6.2	9.4±4.8	10.6±6.9	9.6±5.3	20.3±8.9	2.0±0.8	93.8±25.1	9.7±4.0
*Normal range*			*60–160*	*65–150*	*60–130*	*65–150*	*60–160*	*0*.*75–1*.*2*	*70–140*	*0–5*

ND = not detectable; BS = bleeding score from BAT (bleeding assessment tool).

In the normal-platelet-VWF mild type 1 group, 8 patients belonging to 5 unrelated families were genetically characterized, exploring the whole VWF gene: no VWF mutations were identified in 6 patients (from 3 families), while the other 2 were found heterozygous for the p.C1130F and the new p.R670C mutations. In the low-platelet-VWF mild type 1 VWD group, 32 patients from 17 unrelated families underwent genetic investigations and VWF mutations were identified in all but one: 71% were missense mutations (the most common being the c.7085G>T, p.C2362F—found in 3/17 families) (Table 2), while the other 29% were null mutations, i.e. nonsense nucleotide substitutions or deletions inducing a frameshift with a premature stop codon, the most common being the c.1534-3C>A mutation (in 2/17 families).

**Table 2:** Genetic mutations in the VWD patients studied.

VWD type		Missense	Non sense	Splicing	Deletion/Insertion	Compound
**Mild type 1**	Normal-platelet-VWF	p.R670Cp.C1130F	-	-	-	-
	Low-platelet-VWF	p.Y1584Cp.L1733Pp.P2063Sp.C2362Fp.G2705R	p.C921*p.E2539*	p.L512Pfs*11	p.P812Rfs*31p.Q104Rfs*19p.L757Vfs*22	p.C2627Y; p.C388Wp.C524Y; p.M740I; p.R924Qp.V1279I; p.Q1311*
**Severe type 1**		-	-	-	-	p.L512Pfs*11; p.H2378Afs*?p.L512Pfs*11; p.L512Pfs*11p.L512Pfs*11; p.C584Fp.L512Pfs*11; p.Q706*p.L512Pfs*11; p.C2362Fp.L512Pfs*11; p.H2378Afs; p.R2535*
**Type 3**		-	-	-	-	p.S1338*; p.S1338*p.E244Lfs*211; p.E244Lfs*211p.E244Lfs*211; p.C584F
**Type Vicenza**		p.R1205H	-	-	-	p.M740I; p.R1205H
**Type 2A**	I	p.L1446Pp.R1597W	-	-	-	p.A542G; p.R1374H
	II	p.L1562Pp.I1628Tp.G1609R	-	-	-	-
**Type 2B**	Abnormal multimers	p.R1308Cp.R1306Wp.V1316M	-	-	-	-
	Normal multimers	p.I1372Sp.R1379Cp.R1341W	-	-	-	p.P1266Q; p.R1379C
**Type 2N**	Normal VWF synthesis	p.R854Qp.R760C	-	-	-	p.R760C; p.R854Qp.R854Q; p.R854Q
	Compound orreduced VWF synthesis		-	-	-	p.R854Q; p.G2352_C2360delp.R854Q; p.P812Rfs*31

All mutations are named according to the HGVS (Human Genome Variation Society) recommendations.

**Fig 2:**
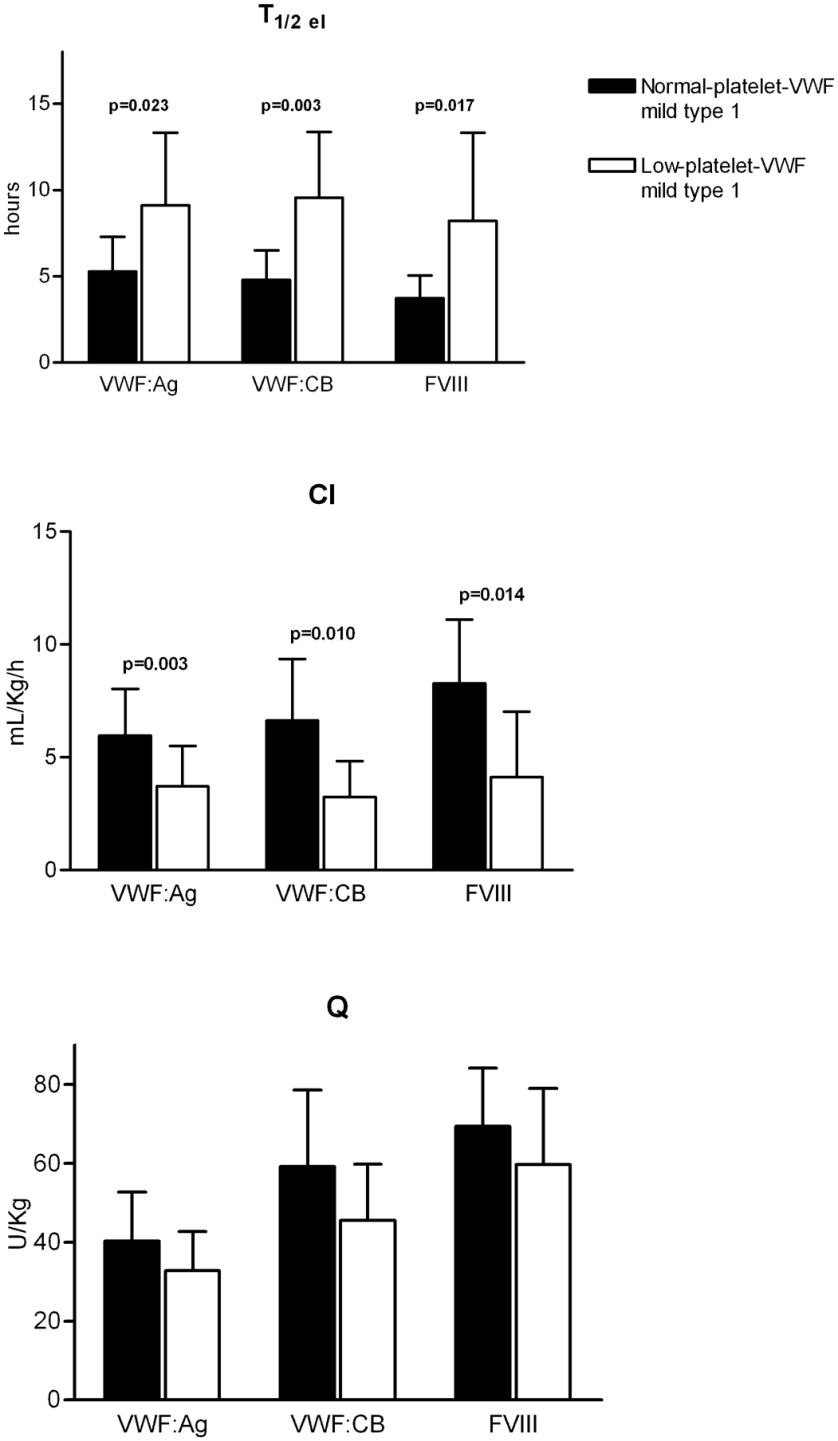
Mean post-DDAVP pharmacokinetic parameters of VWF:Ag, VWF:CB and FVIII in patients with normal-platelet-VWF and low-platelet-VWF mild type 1 VWD. The VWF T_1/2_elimination (T_1/2_el) was statistically shorter, and VWF clearance (Cl) was significantly faster in the former patients than in the latter.

The DDAVP test was performed in 10 normal-platelet-VWF and 8 low-platelet-VWF patients with mild type 1 VWD to examine the contribution of VWF survival in regulating plasma VWF levels: the normal-platelet-VWF patients revealed a statistically lower VWF T_1/2_elimination (T_1/2_el) than the low-platelet-VWF patients (5.3+/-2.0 h vs 9.1+/-4.2 h, p<0.05), and a faster clearance (CL) (5.95+/-2.1 h vs 3.72+/-1.8 h, p<0.005) (Fig 2). The same was true of these patients’ VWF:CB and FVIII values (Fig 2), whereas no significant difference in the quantity (Q) of VWF and FVIII released was observed in any of the patients studied (Fig 2).

#### Severe type 1 VWD

The 8 patients with severe type 1 VWD all had a significantly reduced platelet VWF content (4.8+/-1.0 U/dL), as well as significantly reduced plasma VWF values (5.8+/-0.8 U/dL) (Fig 1). Their FVIII/VWF:Ag ratio appeared to be significantly increased (5.2 +/-0.7) (Table 1). They were all carrying the c.1534-3G>A mutation at homozygous or compound heterozygous level (Table 2) [26,27].

### Type 3

All type 3 patients had platelet VWF levels below 0.10 U/dL and plasma VWF:Ag levels ranging from undetectable to 0.85 U/dL, while their FVIII values were always below 10 U/dL (Table 1). The mutations identified are listed in Table 2.

### Type Vicenza

Ten families with 16 patients who had type Vicenza VWD were studied; all but one were from north-east Italy. Their main hemostatic findings are given in Table 1. Their plasma VWF levels were significantly reduced (10.9+/-3.69 U/dL), while platelet VWF content was normal in all instances (91.1+/-23.6 U/dL) (Fig 1), and the FVIII/VWF:Ag ratio appeared to be higher than normal (2.0+/-0.2) (Table 1). Type Vicenza VWD was first diagnosed on the strength of the low plasma VWF levels, significantly reduced VWF survival, and increased clearance (data not shown) [28]. Type Vicenza patients carried both p.R1205H and p.M740I mutations in 14 cases, and only the p.R1205H mutation in 2 (from 2 unrelated families); the p.R1205H mutation is the one that identifies type Vicenza VWD [29].

### Type 2A

Type 2A VWD is characterized by an abnormal VWF-platelet interaction due to the absence of large VWF multimers. Among the 14 patients studied, 9 were type 2A-I (with an abnormal VWF synthesis and multimerization), and 5 were type 2A-II (carrying mutations that make VWF more susceptible to proteolysis by ADAMTS13). Type 2A-I was characterized by lower plasma VWF:Ag (23.3+/-2.9 U/dL) and platelet (52.6+/-7.3 U/dL) levels, with a more pronounced reduction in VWF:CB and VWF:RCo (13.7+/-2.1 U/dL and 9.9+/-1.1 U/dL, respectively). The FVIII/VWF:Ag ratio was slightly increased (1.8+/-0.2) (Table 3). In type 2A-II, platelet VWF:Ag was normal (mean 80.7+/- 5.1 U/dL), while VWF:Ag was reduced (42.8+/-4.7), and VWF:CB and VWF:RCo were even more so (4.4+/-3.1 U/dL and 19.0+/-2.6 U/dL, respectively) (Table 3), but the FVIII/VWF:Ag ratio was almost normal (Fig 2). The mutations involved in types 2A-I and 2A-II are listed in Table 2.

**Table 3:** Main haemostatic findings of the VWD patients studied characterized by VWF functional abnormalities.

VWD type	Subtype	No subjects/families	VWF:Ag(U/dL)	VWF:CB(U/dL)	VWF:RCo(U/dL)	VWF:FVIIIB(U/dL)	FVIII(U/dL)	FVIII/VWF:Ag	Platelet VWF(U/dL)	RIPA(%)	MADR(mg/mL)	BS
**Type 2A**	I	9/3	23.3±8.6	13.7±6.2	9.9±3.3	17.8±8.7	37.7±10.8	1.8±0.5	52.6±20.6	6.9±3.4	-	10.5±3.7
	II	5/4	42.8±10.4	4.4±5.4	19.0±5.1	46.2±5.7	53.3±6.9	1.3±0.3	80.7±11.5	0	-	5.0±2.6
**Type 2B**	Abnormal multimers	18/9	41.7±16.7	8.3±3.8	16.9±9.2	-	49.4±11.8	1.3±0.4	118.1±42.3	69.5±14.6	0.4±0.1	11.8±4.4
	Normal multimers	10/5	75.2±52.4	75.5±54.8	60.5±47.1	-	74.4±38.6	1.2±0.4	71.9±24.4	78.6±6.8	0.6±0.1	9.7±4.7
**Type 2N**	Normal VWF synthesis	18/11	106±56.2	88.5±35.9	96.2±41.6	59.2±37.2	68.4±28.3	0.7±0.3	122.1±53.0	74.8±21.1	-	3.6±3.0
	Compound or reduced VWF synthesis	3/2	45.4±3.9	46.0±1.6	46.3±4.1	11.6±3.4	29.2±8.1	0.6±0.2	30.4±23.5	72.4±6.8	-	13±3.8
*Normal values*			*60–160*	*65–150*	*60–130*	*65–150*	*60–160*	*0*.*75–1*.*2*	*70–140*	*58–82*	*≥1*.*0mg/mL*	*0–5*

BS = bleeding score from BAT (bleeding assessment tool); RIPA = Ristocetin-induced platelet aggregation at 1.2 mg/mL ristocetin concentration; MADR = minimal aggregating dose ristocetin.

### Type 2B

Type 2B VWD patients were characterized by platelet hyper-responsiveness to ristocetin in the presence of mutations in the VWF A1 domain. Eight patients had no large VWF multimers, while 10 had a full complement of VWF oligomers. Type 2B patients with abnormal VWF multimers had a lower VWF:Ag (41.7+/-3.9 U/dL), and a more pronounced reduction in VWF:CB (8.3+/-0.9 U/dL) and VWF:RCo (16.9+/-2.2U/dL) (Table 3), whatever the mutations they carried (Table 2). Platelet VWF content was normal (118.1+/-10.3 U/dL) in patients with both normal and reduced platelet counts, however. Type 2B patients with a normal multimer pattern had a more complex hemostatic profile, characterized by a concordant reduction in VWF:Ag, VWF:CB and VWF:RCo (Table 3), coinciding with the presence of all VWF multimers. Platelet VWF levels proved more heterogeneous: they were reduced in patients carrying the p.R1379C and p.R1341W mutations (mean 52.36+/-8.9 U/dL), and normal in patients carrying the p.I1372S substitution (mean 115.8+/-20.8 U/dL) [30]. The FVIII/VWF:Ag ratio of type 2B patients with normal multimers was similar to that of normal individuals or patients with type 2B VWD and an abnormal multimer pattern (Table 3). So, while VWF synthesis was normal in patients with an abnormal multimer pattern (consistently with their normal platelet VWF content), type 2B patients with normal multimers usually (though not always) had an impaired VWF synthesis.

### Type 2N

This type of VWD was diagnosed on the grounds of a defective capacity of VWF to bind FVIII, as explored by means of the VWF:FVIIIB test. Among the 21 patients studied, 18 were homozygous or heterozygous for the p.R854Q VWF mutation, 1 was carrying the p.R760C mutation, which induces a persistence of the VWF propeptide that interferes with FVIII binding, 1 was compound heterozygous for the p.R854Q and p.R760C mutations, and 1 was compound heterozygous for the p.R854Q and p.G2352_C2360del mutations. All patients carrying the p.R854Q alone had normal platelet and plasma VWF levels, but if there was a p.R760C mutation, alone or in combination with a p.R854Q mutation, then plasma VWF was reduced (and platelet VWF was not) in the presence of a defective VWF:FVIIIB (Table 3). On the other hand, patients carrying the p.R854Q mutation combined with the p.P812Rfs*31 and p.G2352_C2360del mutations had low levels of both plasma and platelet VWF (Table 3). No significant differences in the FVIII/VWF:Ag ratio emerged between the two groups of patients (Table 3).

## Discussion

The characterization and typing of VWD is a complicated matter and numerous tests are needed to arrive at a precise diagnosis. This study shows that measuring platelet VWF can be helpful in characterizing VWD by revealing when VWF synthesis is impaired.

The VWF abnormalities underlying inherited VWD may concern the synthesis or the functioning of VWF, or combinations of these factors. VWF synthesis is assessed mainly by measuring plasma VWF concentrations, which are the result of the constitutive release of the VWF synthesized in endothelial cells. A reduction in plasma VWF can therefore be interpreted as an expression of its reduced synthesis. Plasma VWF may also be down-regulated due to a shorter VWF survival, however, as seen in type Vicenza VWD, which is characterized by a VWF that is synthesized and stored normally, but subject to an increased clearance [28], and this makes it difficult to interpret the significance of low circulating VWF levels. The DDAVP test also can be used to shed light on VWF synthesis because it induces an acute release of the VWF stored in the Weibel Palade bodies of endothelial cells: since a reduced VWF synthesis will give rise to its reduced storage, the post-DDAVP normalization of plasma VWF may be impaired as a result.

Platelet VWF is synthesized in megakaryocytes and stored in platelet alpha granules by means of processes that are apparently not dissimilar to those involved in endothelial cells. This means that measuring platelet VWF provides information on the processes occurring in endothelial cells. Platelet VWF is rarely measured, but it is a good tool for exploring VWF synthesis because—unlike plasma VWF—it is uninfluenced by environmental factors, especially aging and morbidities; in our experience, platelet VWF does not change significantly with age, whereas circulating VWF does, especially in type 1 VWD patients with mild VWF defects (unpublished data).

In the present study, platelet and plasma VWF levels were almost undetectable in patients with type 3 VWD, confirming that VWF synthesis is negligible or totally lacking in these patients. The picture of type 1 VWD is more complex. Most—but not all—patients with this form showed a correlation between platelet and plasma VWF; this was always true for patients with severe type 1 VWD, but not in those with mild type 1 VWD, some of whom had a normal platelet VWF content, while it was reduced in the others. The two groups of patients identified in this paper as having normal-platelet-VWF and low-platelet-VWF mild type 1 VWD had no statistically significant differences in their circulating VWF levels. The patients with a normal platelet VWF content included both single cases and familial clusters, which confirms that their hemostatic picture is genetically determined. These patients’ bleeding scores were normal or slightly increased (with no significant differences between the two sub-groups). The difference in platelet VWF content therefore does not seem to significantly affect a patient’s tendency to bleed.

All but one of the genetically investigated low-platelet-VWF mild type 1 patients carried VWF mutations, whereas only 2 of the 8 normal-platelet-VWF mild type 1 patients revealed VWF mutations (p.C1130F and p.R670C). This raises the question of why plasma VWF is reduced in these patients despite their normal VWF synthesis. An answer comes from the DDAVP test, which showed that VWF survival and clearance were respectively lower and higher in mild type 1 VWD patients with normal platelet VWF than in those with low platelet VWF. It is easy to explain why this happens in the patient carrying the p.C1130F mutation (known to be associated with a shorter VWF half-life) [31], but not in the others—especially those who apparently have no mutations in the coding portion of their VWF gene. It might be tentatively advanced that these patients have unknown factors outside the VWF molecule that regulate VWF clearance. In addition, measuring platelet VWF also seems useful for predicting response to DDAVP when it has to be used for therapeutic purposes. It has been clearly demonstrated by different groups that type 1 VWD patients with low platelet VWF levels [32] respond to DDAVP less well than patients with a normal platelet VWF content [16, 33].

Type Vicenza is probably one of forms of VWD in which measuring platelet VWF is particularly useful. Circulating VWF levels are reduced in these patients, with values sometimes below 10 U/dL (as in severe type 1 VWD), while their platelet VWF content is normal. At our laboratory, these two divergent features are considered the hallmark of type Vicenza patients because, to the best of our knowledge, no other forms of VWD feature this marked discrepancy; this finding consequently prompts a search for the type Vicenza mutation (p.R1205H) [29].

Measuring platelet VWF is similarly useful in type 2A VWD, which is characterized by an abnormal platelet-VWF interaction due to a shortage of large VWF multimers. Two subtypes of type 2A VWD have been described: type 2A-I with a low platelet VWF content, and type 2A-II with a normal platelet VWF content [34]. It may be difficult to distinguish between the two forms, and measuring platelet VWF can be very helpful in this setting. Drawing this distinction is by no means irrelevant because patients with type 2A-I VWD generally have a more pronounced bleeding tendency than those with type 2A-II, as demonstrated by their BS.

Two main pictures can be seen in type 2B VWD as well, i.e. patients lacking in circulating large VWF multimers and those with a normal multimer pattern. In both cases, their VWF has a greater affinity for platelet GPIb. Platelet VWF levels are normal in the former, and often (but not always) lower than normal in the latter. Measuring platelet content is probably less useful in this setting than in the previously-mentioned types of VWD because in type 2B VWD it is important to ascertain the greater affinity of VWF for platelets and check for the presence or absence of large VWF multimers. Establishing patients’ platelet VWF levels is still useful, however, for characterizing the VWD phenotype.

Platelet VWF is always normal in type 2N VWD, unless patients simultaneously carry a second mutation that impairs VWF synthesis, such as the p.P812Rfs*31 and p.G2352_C2360del mutations found in our cohort. This means that measuring platelet VWF in cases of type 2N VWD can help to establish whether any other mutations are involved, particularly considering that recessive quantitative defects may not be very obvious if only plasma VWF is measured (as discussed above). This applies to other forms of VWD too, when the phenotype does not neatly fit the canonical criteria.

Other tools are currently used to identify a defective VWF synthesis, the most commonly adopted being the FVIII/VWF:Ag ratio [35,36]: a higher ratio is suggestive of a reduction in VWF synthesis, while a normal ratio points to VWF alterations being attributable to a shorter VWF survival. This distinction seems to apply in our patients too—with the exception of type Vicenza VWD patients, whose FVIII/VWF:Ag ratio increases despite their main defect being associated with a marked reduction in VWF survival. For other forms of type 1 VWD, a correlation is apparent between a rising FVIII/VWF:Ag ratio and falling platelet VWF levels, since patients with severe type 1 VWD had the most pronounced increase in their FVIII/VWF:Ag ratio, while it was nearly normal in patients with normal-platelet-VWF mild type 1 VWD. The same applies to VWD types 2A and 2B, though the increase in the FVIII/VWF:Ag ratio in the forms associated with a defective VWF synthesis is negligible, and consequently less informative. Combining the FVIII/VWF:Ag ratio with platelet VWF levels could therefore facilitate the demonstration of a defective VWF synthesis.

In conclusion, measuring platelet VWF is an excellent way to study VWD for several reasons: it enables a distinction to be drawn between quantitative and qualitative VWF defects; it helps to identify the affected members in the families of patients with type 1 VWD, especially when their plasma VWF levels are not informative; it distinguishes between cases of mild type 1 VWD with normal as opposed to low platelet VWF levels; it grades the severity of a defective VWF synthesis; it identifies cases of type Vicenza VWD; it distinguishes type 2A-I from type 2A-II disease; it better characterizes type 2B VWD; it identifies type 2N patients who carry quantitative VWF mutations too; it predicts which patients will respond to DDAVP, and also the bleeding risk of VWD patients, especially for those carrying a quantitative VWF defect.
